# *In Vitro* Evaluation of the Antibacterial Activity of the Peptide Fractions Extracted from the Hemolymph of *Hermetia illucens* (Diptera: Stratiomyidae)

**DOI:** 10.3390/insects14050464

**Published:** 2023-05-15

**Authors:** Carmen Scieuzo, Fabiana Giglio, Roberta Rinaldi, Marilena E. Lekka, Flora Cozzolino, Vittoria Monaco, Maria Monti, Rosanna Salvia, Patrizia Falabella

**Affiliations:** 1 Department of Sciences, University of Basilicata, Via dell’Ateneo Lucano 10, 85100 Potenza, Italy; carmen.scieuzo@unibas.it (C.S.); fabiana.giglio@unibas.it (F.G.); roberta.rinaldi@unibas.it (R.R.); 2 Spinoff XFlies s.r.l., University of Basilicata, Via dell’Ateneo Lucano 10, 85100 Potenza, Italy; 3 Laboratory of Biochemistry, Department of Chemistry, University of Ioannina, 45110 Ioannina, Greece; mlekka@uoi.gr; 4 Department of Chemical Sciences, University of Naples Federico II, 80126 Naples, Italy; flora.cozzolino@unina.it (F.C.); monacovi@ceinge.unina.it (V.M.); montimar@unina.it (M.M.); 5 CEINGE Advanced Biotechnologies, University of Naples Federico II, 80145 Naples, Italy

**Keywords:** antibiotic resistance, black soldier fly, AMPs, *Escherichia coli*, *Micrococcus flavus*

## Abstract

**Simple Summary:**

Antibiotic resistance is a worldwide social and health crisis. The search for therapeutic alternatives, including the use of antimicrobial peptides (AMPs), is critical. AMPs are small molecules synthesized by a wide range of living organisms. Microbiological and mass spectrometric techniques were used to examine peptides in the hemolymph of larvae of the scavenger insect *Hermetia illucens* (Diptera, Stratiomyidae) after infection with *Escherichia coli* or *Micrococcus flavus*, as well as uninfected larvae, used as control. Microbiological assays allowed us to confirm antimicrobial activity of *H. illucens* AMPs, while via mass spectrometry we identified a set of 33 AMPs, expressed in different conditions: 20 AMPs were expressed in all the analyzed conditions, while 13 were differentially expressed after Gram negative or Gram positive bacterial challenge. Differentially expressed AMPs may be responsible for a more specialized action.

**Abstract:**

Antimicrobial peptides (AMPs) are a chemically and structurally heterogeneous family of molecules produced by a large variety of living organisms, whose expression is predominant in the sites most exposed to microbial invasion. One of the richest natural sources of AMPs is insects which, over the course of their very long evolutionary history, have adapted to numerous and different habitats by developing a powerful innate immune system that has allowed them to survive but also to assert themselves in the new environment. Recently, due to the increase in antibiotic-resistant bacterial strains, interest in AMPs has risen. In this work, we detected AMPs in the hemolymph of *Hermetia illucens* (Diptera, Stratiomyidae) larvae, following infection with *Escherichia coli* (Gram negative) or *Micrococcus flavus* (Gram positive) and from uninfected larvae. Peptide component, isolated via organic solvent precipitation, was analyzed by microbiological techniques. Subsequent mass spectrometry analysis allowed us to specifically identify peptides expressed in basal condition and peptides differentially expressed after bacterial challenge. We identified 33 AMPs in all the analyzed samples, of which 13 are specifically stimulated by Gram negative and/or Gram positive bacterial challenge. AMPs mostly expressed after bacterial challenge could be responsible for a more specific activity.

## 1. Introduction

Infectious diseases have always been one of the major threats to human and animal health and a major cause of morbidity and mortality [1,2]. The discovery of antibiotics was a powerful tool to support medicine for the treatment of bacterial infections and the associated complications. The progressive misuse of antibiotics has unfortunately favored the selection and spread of resistant populations of bacterial agents [3,4]. The drug-resistance phenomenon has a heavy impact on the world community [5,6]. Following the development of antibiotic-resistant bacterial strains and the reduced availability of effective antibiotics, a need to identify new molecules to be used for the development of alternative therapies is growing [7,8]. Antimicrobial peptides (AMPs) are small cationic molecules, containing from 10 to 50 amino acids, able to selectively bind the membranes of bacteria, disrupting them and inducing cell death [9,10]. They constitute one of the first lines of defense of organisms against a great variety of external agents [11,12].

Several characteristics of AMPs make them particularly interesting as potential therapeutic tools, as they manifest synergies with the acquired immune system [13] and demonstrate specificity towards prokaryotic cells [14]: due to their positive charge, the AMPs establish an electrostatic interaction with the surface of the pathogens that exposes a net negative charge [15,16]; they demonstrate broad-spectrum activity against viruses, bacteria and fungi [17], kill rapidly (99.9% of bacteria treated in 20 min) [18], show synergies with conventional antibiotics [19,20], are effective against antibiotic-resistant bacteria and they do not cause the selection of new resistant mutants, as they act on bacterial cell membranes with mechanisms different from those of common drugs [21].

AMPs are essential components of the first line of defense systems of bacteria, plants and animals, including mammals [22,23]. Their production within various organisms is specific and can occur in a constitutive manner or can be induced in response to an external insult by pathogens [23,24]. Among invertebrates, insects, with more than one million species described, represent a source of great interest. AMPs are part of the humoral immune response of insects [25,26,27]. In holometabolous species AMPs are biosynthesized mainly in the fat body and transferred into the hemolymph [28] from which they can spread and act throughout the organism; in heterometabolous species, they are produced by haemocytes and secreted into the hemolymph following infection [28,29,30,31]. One of the most appealing insects for the AMP production is the Diptera *Hermetia illucens* (Linnaeus, 1758), commonly known as black soldier fly. *H. illucens* larvae, attracted by specific volatile organic compounds [32], feed on decaying organic substrates of vegetable and animal origin [33,34,35], converting them into a high-value biomass made up of proteins and lipids that can be used in a variety of applications, including feed, energy and cosmetics industry [36,37], as well as to extract high-value compounds for application in biomedical and pharmaceutical fields [38,39,40]. Because of their nutritional substrates, they are exposed to a high and constant concentration of pathogenic microorganisms such as bacteria and fungi present in these substrates [41]. In order to survive, larvae have developed a powerful immune system, with high production of AMPs [42]. These molecules can be constitutively expressed, or their expression can be strongly influenced both by the microorganisms they come into contact with and by the composition of the diet itself [43]. The analysis of one of the *H. illucens* transcriptomes allowed the identification of 57 putatively active AMPs, also characterized by bioinformatic tools, belonging to different classes (defensins, cecropins, attacins, diptericins, knottin-like, stomoxyn-like, alo-1 like and lysozyme) [44,45]. Recent studies have also highlighted the potential antimicrobial activity of some *H. illucens* AMPs against *Staphylococcus aureus*, methicillin-resistant *S. aureus* and *Pseudomonas aeruginosa* [46,47]. *H. illucens* can potentially be an excellent source of new compounds to use alone or in synergy with common antibiotics, especially against resistant strains [48,49]. The aim of this work was to identify the AMPs in *H. illucens* hemolymph, both from uninfected larvae and from larvae infected with *Escherichia coli* (Gram negative) or *Micrococcus flavus* (Gram positive). The peptide component isolated from the hemolymph was analyzed via preliminary microbiological tests and via mass spectrometry to specifically identify constitutive and induced peptides, differentially expressed after bacterial challenge. These peptides could have potential application in biomedical and pharmacological fields, to make an innovative contribution to counteract the antibiotic-resistance issue.

## 2. Materials and Methods

### 2.1. Hermetia illucens Rearing

*Hermetia illucens* larvae were provided by Xflies s.r.l (Potenza, Italy). After egg hatching, larvae were fed on a standard Gainesville diet (30% alfalfa, 50% wheat bran, 20% corn meal) [50] at 70% moisture under controlled conditions of temperature (27 ± 1.0 °C), relative humidity (70% ± 5%) and photoperiod (12L:12D (h)) [35].

### 2.2. H. illucens Larval Infection and Hemolymph Collection

*Escherichia coli* (Gram negative, LMG:2092 strain) and *Micrococcus flavus* (Gram positive, DSM 19079) were incubated in 10 mL of Luria Bertani (LB) broth (1% tryptone, 0.5% yeast extract, 0.5% NaCl), at 37 °C for 24 h, under shaking. A total of 1 mL of each bacterial culture was inoculated into a fresh LB broth, incubated at 37 °C and used for the experiment once the optical density (OD) at 600 nm reached 1. Last instar larvae of *H. illucens* were firstly washed with sterile water and then infected via a capillary dipped into the cell suspension of *E. coli* or *M. flavus* [51,52] in order to stimulate the production of different antimicrobial peptides (AMPs). Following the bacterial challenge, larvae were left in a controlled chamber at 27 °C for 24 h. A group of uninfected larvae was used as control. For each treatment, 100 larvae were used. To facilitate the spill of hemolymph, larval abdomens were punctured by a sterile capillary and the hemolymph from infected and uninfected larvae was collected, using a pipette (Gilson, Middleton, WI, USA), in ice-cold tubes, containing a fixed-minimum quantity of l-ascorbic acid (0.015 g) (Merck Millipore, Burlington, MA, USA), to prevent hemolymph melanization. To recover only the plasma and remove the cellular components, the extracted hemolymph was subjected to centrifugation at 10,000 rcf for 5 min at 4 °C. The recovered supernatant (cell-free hemolymph) was stored at −80 °C until use.

### 2.3. Peptide Fraction Precipitation by Organic Solvents

In order to separate the putative AMPs in the hemolymph from the higher molecular weight proteins, the plasma recovered from both uninfected and infected larvae was subjected to a precipitation protocol with methanol (Merck Millipore, Burlington, MA, USA), acetic acid (Merck Millipore, Burlington, MA, USA) and water in a 90:1:9 *v/v* ratio. Sample and solvent were mixed in a 1:9 *v/v* ratio. The sample was centrifuged for 45 min at 16,000 rcf at 4 °C. The obtained supernatant, containing compounds with a molecular weight lower than 30 kDa, was then vacuum dried to remove the organic solvents and resuspended in a volume of sterile water equal to the original plasma volume. To remove possible traces of lipids that could be co-extracted due to the use of methanol, a further treatment with hexane was performed. Specifically, an equal volume of hexane (Merck Millipore, Burlington, MA, USA) was added to each extract. The samples were vortexed and centrifuged at 16,000 rcf for 20 min at 4 °C [53]. The upper fraction, possibly containing lipids, was removed and stored for the subsequent evaluation via antibiogram assay (Section 2.5). All samples were subsequently stored at 4 °C until next use.

### 2.4. Protein Quantification via Bradford Assay

The concentrations of all samples were quantified with Bio-Rad Protein Assay, Dye Reagent Concentrate (Bio-Rad, Hercules, CA, USA), according to the Bradford method [54]. To calculate the concentration of the proteins of interest, a standard calibration using known concentrations of the Bovine Serum Albumin (BSA) protein (Merck Millipore, Burlington, MA, USA) was set up. The absorbance of the samples was measured at a wavelength of 595 nm using a spectrophotometer (Thermo Scientific, Waltham, MA, USA).

### 2.5. Evaluation of the Antibacterial Activity of Hemolymph via Antibiogram Assay

The *in vitro* evaluation of the antimicrobial activity of hemolymph extracts was carried out via antibiogram (agar diffusion test), using a solution of LB-Agar. A colony of *E. coli* and a colony of *M. flavus* were transferred each to 10 mL of LB and incubated overnight at 37 °C, under shaking. The bacterial culture was uniformly distributed on the agar-containing plates, using a cotton swab. Following its adsorption, 5 μL of each sample, the peptide fractions of the hemolymph extracted from infected and uninfected larvae, was dispensed onto the plate. As a negative control, 5 μL of sterile water was used. All tests were performed in triplicate, incubating the plate overnight at 37 °C.

### 2.6. Evaluation of the Hemolymph Antibacterial Activity via Bioautography (SDS Gel Overlay Method) Experiment

The antibacterial activity of the peptide fraction recovered from the plasma of infected and uninfected larvae was also evaluated via a bioautography experiment [55]. Briefly, two polyacrylamide gels were prepared (4% stacking, 12% running); one of the two gels was stained with a solution of Blue Coomassie (Merck Millipore, Burlington, MA, USA) in order to visualize the bands corresponding to the peptide samples, while the second gel was washed with Triton X-100 (Bio Rad, Hercules, CA, USA) at 2.5% for 1 h to remove the SDS and with Tris-HCl 50 mM pH 7.5 for 2 h to allow the renaturation of the peptides; finally, the gel was incubated in LB culture medium for 1 h. At the end of the incubation in LB, solid nutrient LB-agar culture medium (0.7%) containing *E. coli* or *M. flavus* cells was transferred onto the gel and incubated for 24 h at 37 °C. For each experimental condition, 20 μL of sample was loaded.

### 2.7. Evaluation of the Hemolymph Antibacterial Activity via Microdilution Assay

For the microdilution assay, performed against both *E. coli* and *M. flavus* cultures, the major quantity used in the antibiogram assay was used as a starting quantity (4.5 µg) that was subsequently subjected to serial dilution for a total of 6 serial dilutions (2.24 µg, 1.13 µg, 0.56 µg, 0.28 µg, 0.14 µg). Experimentally, cultures of both *E. coli* and *M. flavus* were seeded in 96-well plates (1 × 10^6^ cells per well) and treated with the serial dilutions, reaching a final volume of 200 μL. Wells containing water and culture alone were used as controls. Plates were incubated at 37 °C to allow bacterial growth for 24 h of incubation, the absorbance of the samples under examination was measured using a spectrophotometer (Thermo Scientific, Waltham, MA, USA) at a wavelength of 600 nm. The experiments were carried out in three technical replicates for each of the three biological replicates. Results were reported as percentage of bacterial culture treated in different conditions compared to culture alone (control), whose value was considered as 100%.

### 2.8. SDS-PAGE and In Situ Hydrolysis

The peptide fraction extracted from *H. illucens* larvae infected with *E. coli*, *M. flavus* and from uninfected larvae (control) was fractionated via sodium dodecyl sulfate-polyacrylamide gel electrophoresis (SDS-PAGE). In detail, at 15 µL for each protein extract, the loading buffer 1X, composed of 2% SDS (Bio-Rad, Hercules, CA, USA), 50 mM TRIS-HCl pH 6.8 (Merck Millipore, Burlington, MA, USA), 10% Glycerol (Merck Millipore, Burlington, MA, USA) and bromophenol blue (Bio-Rad, Hercules, CA, USA), was added, and they were separated on a 20% SDS-PAGE gel. After the run, the gel was stained with GelCode™ Blue Safe Protein Stain (Thermo Fisher Scientific, Waltham, MA, USA) and destained with Milli-Q water. A total of 3 bands for each condition (*E. coli*, *M. flavus,* control) were cut and *in situ* hydrolyzed with trypsin as previously described [56]. Peptide mixtures were extracted in 0.2% formic acid (HCOOH) (Merck Millipore, Burlington, MA, USA) and acetonitrile (ACN) (Merck Millipore, Burlington, MA, USA) and vacuum dried via a SpeedVac System (Thermo Fisher Scientific, Waltham, MA, USA).

### 2.9. LC-MS/MS Analysis and Protein Identification

Each peptide mixture was dissolved in 10 μL of 0.2% HCOOH (Merck Millipore, Burlington, MA, USA) and analyzed via nano LC-MS/MS on an LTQ Orbitrap mass spectrometer (Thermo Fisher Scientific, Waltham, MA, USA) coupled to a nanoLC system nano Easy II. Each peptide mixture was concentrated and desalted onto a trapping column (C18 Easy Column L = 2 cm, ID = 100 mm, Nano Separations, Nieuwkoop, the Netherlands), and then fractionated on a C18 reverse-phase capillary column (C18 Easy Column L = 20 cm, ID = 7.5 µm, 3 µm, (Nano Separations, Nieuwkoop, The Netherlands) with a flow rate of 250 nL/min. The gradient used for peptide elution ranged from 10% to 60% of eluent B in 69 min [57]. Eluents A and B have the following composition: 2% ACN LC-MS grade and 0.2% HCOOH, and 95% ACN LC-MS grade and 0.2% HCOOH, respectively. The MS/MS method was set up in a data-dependent acquisition mode (DDA), with a full scan ranging from 300 to 1800 *m/z* range, followed by fragmentation in CID modality of the top 5 ions (MS/MS scan) selected by intensity and charge state (+2, +3, +4 charges), and applying a dynamic exclusion time of 40 s [58]. The peak list generated was uploaded in Mascot software (version 2.4.0) and research was performed by using the in-house database named the “*Hermetia illucens* database”. The parameters for protein identification were as follows: “trypsin” as enzyme with at least one missed cleavage, “carbamidomethyl” as a fixed modification, “oxidation of Met” and “pyro-Glu at N-term if Gln” as variable modifications, 0.6 Da as MS/MS tolerance and 10 ppm as peptide tolerance. Scores threshold of matches for MS/MS data was fixed at 10 for all peptides.

### 2.10. Statistical Analysis

All experiments were performed in triplicates (three independent biological replicates) and results were expressed as means ± standard error. Data were analyzed via GraphPad Prism 6.0 software (GraphPad Software, Inc., La Jolla, CA, USA) using one-way analysis of variance (ANOVA) followed by Bonferroni *post hoc* test.

## 3. Results

### 3.1. Evaluation of Sample Concentration

The concentration of the samples obtained following precipitation with organic solvents was evaluated via the Bradford assay. The values obtained are shown in the following table (Table 1):

### 3.2. Evaluation of the Antibacterial Activity of Peptide Fraction of Hemolymph via Antibiogram Assay

**Figure 1:**
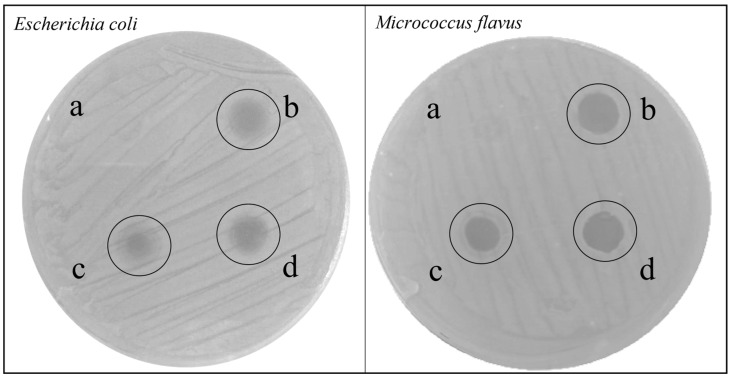
Agar diffusion test of peptide fractions obtained via precipitation with organic solvents, performed against *E. coli* (on the left) or *M. flavus* (on the right). (**a**) H_2_O, negative control; (**b**) peptide fraction from larvae infected with *E. coli*; (**c**) peptide fraction from uninfected larvae; (**d**) peptide fraction from larvae infected with *M. flavus*. The experiments were carried out in triplicate (three independent biological replicates).

The peptide fractions recovered following precipitation with methanol/acetic acid/water (90:1:9 *v/v* ratio) of the plasma extracted from uninfected larvae and from larvae infected with *E. coli* or *M. flavus*, were first analyzed via agar diffusion test to evaluate their antibacterial effect against *E. coli* and *M. flavus*. The test performed both against *E. coli* and *M. flavus* revealed the presence of an inhibition zone, in correspondence with all the analyzed samples (Figure 1). Differences were detected against the two analyzed strains: halos were wider (Table 2) and well defined in the plate with *M. flavus*, compared to the *E. coli* plate, in which the bacterial growth was not completely inhibited, as demonstrated by a patina of bacterial cells on the halo surface.

### 3.3. Evaluation of the Antibacterial Activity of Peptide Fraction of the Hemolymph via Bioautography (SDS Gel Overlay Method) Assay

**Figure 2:**
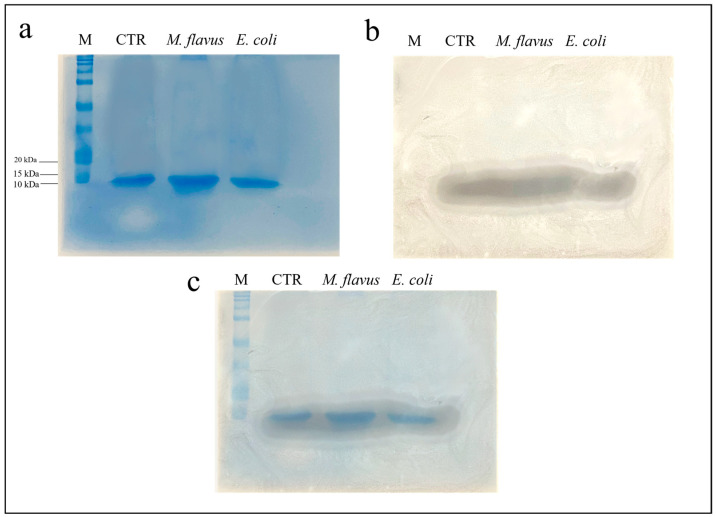
SDS-PAGE (**a**) and bioautography (**b**) performed against *E. coli* of the samples obtained following precipitation with organic solvents. In (**c**), an overlay of the previous images is presented. M = marker, “All Blue Standards Biorad” (Biorad, Hercules, CA, USA). CTR = peptide fraction from uninfected larvae; *M. flavus* = peptide fraction from larvae infected with *M. flavus*; *E. coli* = plasma from larvae infected with *E. coli*. The experiments were carried out in triplicate (three independent biological replicates).

**Figure 3:**
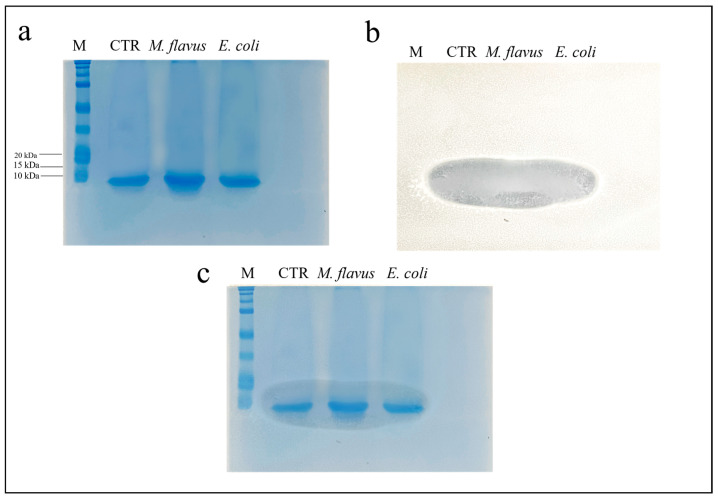
SDS-PAGE (**a**) and bioautography (**b**) performed against *M. flavus* of the samples obtained following precipitation with organic solvents. In (**c**), an overlay of the previous images is presented. M = marker, “All Blue Standards Biorad” (Biorad, Hercules, CA, USA). CTR = plasma from uninfected larvae; *M. flavus* = plasma from larvae infected with *M. flavus*; *E. coli* = plasma from larvae infected with *E. coli*. The experiments were carried out in triplicate (three independent biological replicates).

An electrophoretic analysis of the infected and uninfected samples, treated with methanol, acetic acid and water in a 90:1:9 ratio *v/v* was performed. Three identical gels (12% acrylamide) were prepared and at the end of the electrophoretic run, one of the gels was stained with Coomassie Blue, while on the other gels a bioautography test against *E. coli* and *M. flavus* was performed. Results in Figure 2a and Figure 3a show the presence of low molecular weight bands, around 10 kDa. Figure 2b and Figure 3b show an inhibition zone in correspondence with low molecular weight bands relative to the peptide fraction obtained following precipitation of the plasma extracted from all samples, and tested against *E. coli* and *M. flavus*, respectively. Figure 2c and Figure 3c show the overlay between the gel and the inhibition zone observed on bioautography, to confirm that the obtained inhibition comes from peptides around 10 kDa.

### 3.4. Evaluation of the Biological Activity of the Peptide Fractions via Liquid Microdilution Assays

**Figure 4:**
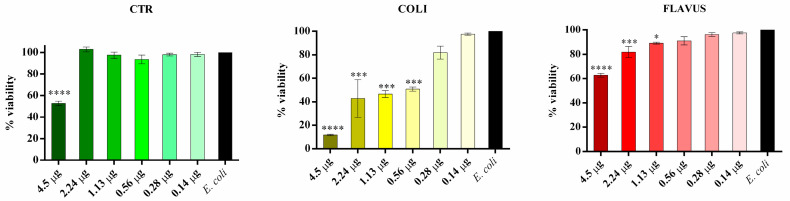
Microdilution assay against *E. coli* performed with the peptide fractions obtained via precipitation with organic solvents. CTR = peptide fractions from uninfected larvae; COLI = peptide fractions from larvae infected with *E. coli*; FLAVUS = peptide fractions from larvae infected with *M. flavus*. The black bars represent the untreated *E. coli* cell culture. Results are presented as percentage of viability of bacterial culture treated in different conditions compared to culture alone (control), whose value was considered as 100%. Data are expressed as means ± standard error of three independent biological replicates and statistical significance was evaluated with one-way ANOVA followed by Bonferroni *post hoc* test (* *p* < 0.1, *** *p* < 0.001, **** *p* < 0.0001).

**Figure 5:**
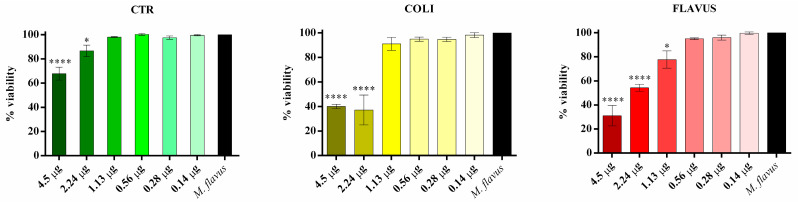
Microdilution assay against *M. flavus* performed with the peptide fractions obtained via precipitation with organic solvents. CTR = peptide fractions from uninfected larvae; COLI = peptide fractions from larvae infected with *E. coli*; FLAVUS = peptide fractions from larvae infected with *M. flavus*. The black bars represent the untreated *M. flavus* cell culture. Results are presented as percentage of viability of bacterial culture treated in different conditions compared to culture alone (control), whose value was considered as 100%. Data are expressed as means ± standard error of three independent biological replicates and statistical significance was evaluated with one-way ANOVA followed by Bonferroni post hoc test (* *p* < 0.1, **** *p* < 0.0001).

Starting from the qualitative results obtained by agar diffusion and bioautography tests, microdilution assays against *E. coli* (Figure 4) and *M. flavus* (Figure 5) were performed.

All the analyzed samples are able to inhibit *E. coli* cell growth, although with different minimum inhibitory concentrations (MICs) and percentage of reduction. Indeed, sample control can inhibit cell growth by 42%, exclusively at the highest quantity tested (4.5 µg). The MIC of peptide fractions obtained from larvae infected with *E. coli* is 0.56 µg, with a reduction in cell growth of 50%, while as concerns peptide fractions obtained from larvae infected with *M. flavus* MIC is 1.13 µg, with a reduction in cell growth of 11%. The highest quantity of peptides obtained from larvae infected with *E. coli* is able to reduce the growth by 89%, while the highest quantity of peptides obtained from larvae infected with *M. flavus* is able to reduce the growth by 32%.

All the examined samples may prevent *M. flavus* cell development, albeit at varying MICs and reduction rates. Indeed, the highest tested quantity for the sample control (4.5 µg) can inhibit cell growth by 33%, whereas the MIC value (2.24 µg) inhibits the 14% growth. Differently to the MIC against *E. coli*, in the peptide fractions obtained from larvae infected with *E. coli* this value is 2.24 µg, with a reduction in cell growth of 63%, the same percentage obtained by the highest quantity used. As concerns peptide fractions obtained from larvae infected with *M. flavus*, the MIC value is 1.13 µg, with a reduction in cell growth of 33%. The highest quantity of this peptide fraction is able to reduce the growth by 69%.

### 3.5. Mass Spectrometry Analysis

After SDS analysis, bands were *in situ* hydrolyzed via trypsin, and the peptide mixtures were analyzed via LC-MS/MS. The raw data from mass spectrometry analysis were converted to mgf files and then inserted into the MASCOT software for protein identification. The protein database used consists of contigs containing putative protein sequences derived from *H. illucens* transcriptomes. Six putative protein sequences, each with a single reading frame, are presented for each contig. In Table S1a–d, the identified peptides are presented including the following information: experimental *m/z* value of the peptide, experimental mr value, the mascot score, the sequence of identified peptides, the contig code, the amino acid sequence frame (in red the peptides found by LC-MS/MS) and the frame number of the transcriptomic sequence obtained with SEQtools that match with the LC-MS/MS.

**Figure 6:**
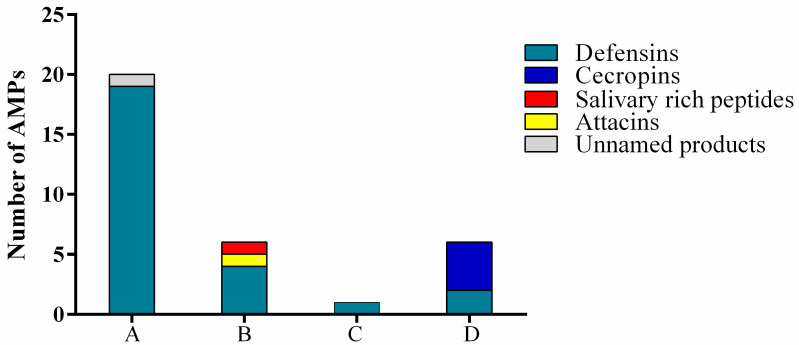
Number and classes of AMPs identified via the LC-MS/MS in different experimental conditions: A = peptides identified both in infected and uninfected larvae; B = peptides identified in larvae infected with *E. coli* or *M. flavus*; C = peptide identified exclusively in larvae infected with *E. coli*; D = peptides identified exclusively in larvae infected with *M. flavus.*

We identified 33 AMPs (Figure 6): 20 expressed in all the analyzed conditions, 6 absent in control and expressed only after infection with *E. coli* or *M. flavus*, 1 differentially expressed after infection of *E. coli* and 6 differentially expressed after infection with *M. flavus.* The 6 AMPs differentially expressed after the infection of both bacteria were 4 defensins, 1 attacin and 1 uncharacterized protein; the AMPs expressed after *M. flavus* infection were 4 cecropins and 2 defensins, while the differentially AMP expressed after *E. coli* infection was a defensin.

## 4. Discussion

In recent decades, the excessive and inappropriate use of antibiotics in human and veterinary medicine has contributed to an increase in the natural selection of resistant bacteria and a decrease in drug efficacy [59,60]. Few classes of antibiotics are now effective against some multi-resistant pathogenic bacteria and the worldwide spread of resistance genes is considered a scenario of extreme emergency [61]. For this reason, the search for new molecules with antibacterial activity represents one of the major current challenges for the scientific community. Antimicrobial peptides (AMPs) represent an excellent alternative to modern antibiotics [62,63]. AMPs are small molecules positively charged that selectively interact with the negatively charged bacterial surface [64]. One of the richest sources of AMPs is represented by the class of insects which is characterized by the large quantity and the diversity of its molecules and processes. Insects are organisms extremely well adapted to diverse habitats, primarily due to their innate immune system, which provides them with a range of cellular and humoral responses against microorganisms [65]. Moreover, insects can also feed on substances with different levels of contaminations, so they synthesize AMPs to fight such infections and survive in dangerous conditions [66]. AMPs extracted from insects have the potential to fight the microorganisms that act as hazards to human health [66,67]. One of the most interesting insect species is Diptera *Hermetia illucens*, which is able to produce a number of AMPs, far superior to that of other insects [44]. The wide spectrum of produced AMPs is directly related to the remarkable variety of substrates on which the larva feeds on. The present work is part of a broader project of identification and structural and functional characterizations of the AMPs produced by *H. illucens* larvae, in order to use them as highly innovative antimicrobial molecules. In this work, we focused on the *in vitro* evaluation of the antimicrobial activity of the peptide fraction of the hemolymph of *H. illucens*, following infection with the Gram negative bacterium *Escherichia coli* or the Gram positive *Micrococcus flavus*, via microbiological tests performed against *E. coli* and *M. flavus* themselves. Following the precipitation of the peptide fraction, a further extraction step in hexane was performed to ensure the absence of any traces of lipids, which have antimicrobial activity due to the presence of lauric acid [37]. As shown in Figure S1, no activity was detected in the upper fraction of the hexane extract, the fraction that should contain lipid traces, demonstrating that the antimicrobial activity detected is exclusively attributable to the AMPs. From our data, it is possible to highlight that AMPs produced by *H. illucens* are effective both against Gram positive and Gram negative bacteria, and that the expression of some AMPs can be induced following the stimulation by specific bacteria. Although the microbiological analyses (antibiogram, bioautography and microdilution assay) were the starting point of our experiments, for the identification of constitutive and inducible AMPs and the differential expression after the bacterial challenge with Gram negative and Gram positive bacteria, a mass spectrometry analysis was also performed. With a combined transcriptomic and proteomic approach, we identified 20 AMPs constitutively expressed, whose expression could increase after bacterial infection, and 13 inducible. The bacterial infection, indeed, stimulates the expression of specific peptides. Both *E. coli* and *M. flavus* induced the expression of 6 AMPs (defensins, attacins and cysteine-rich peptide), while a defensin was induced specifically by *E. coli*, cecropins and defensins by *M. flavus*. As expected, the most detected AMPs were defensins [45].

Usually, insect defensins are more active against Gram positive bacteria such as *S. aureus* [47] or *Bacillus subtilis* [68]; however, some of them also exhibit antimicrobial activity against Gram negative bacteria, in particular *E. coli* [47,69]. Defensin expression can be induced by Gram negative [70,71,72] or positive bacteria [70,73,74], as also recorded via experiments carried out in this work.

Cecropins, α-helical AMPs, are indiscriminately active against Gram negative bacteria, such as *E. coli*, *Klebsiella pneumoniae*, *Salmonella typhimurium* and *Pseudomonas aeruginosa* [75,76,77], or Gram positive bacteria, such as *Staphylococcus* and *Bacillus* species [75,76,77]. Their expression can be induced by both Gram positive and negative bacteria [78]: for example, in Lepidoptera, different microbial infections result in different patterns of cecropin gene expression, indicating that various signaling pathways can contribute to the same immune gene expression.

The results obtained from our experiments suggested that, depending on the bacteria used for the infection, different AMPs could be induced, as previously reported for *Drosophila melanogaster* [79,80], *Diatraea saccharalis* [65], *Galleria mellonella* [81], *Rhynchophorus ferrugineus* [82].

In Rocha *et al*., *D. saccharalis* larvae were challenged with *E. coli* and *B. subtilis*. The infection with the Gram positive bacteria induced more pronounced antibacterial activity (evaluated via antibiogram against *B. subtilis*) corresponding to an increase in the expression of 2 AMPs, a defensin and an attacin. Infection with Gram negative bacterium, on the other hand, induced an exclusive increase in the levels of the attacin [65].

In Mak et al. [81], *Galleria mellonella* larvae were challenged with *E. coli* and *M. luteus* and tested against *E. coli*, finding stronger activity by larvae challenged with the Gram negative bacterium. Then differentially expressed peptides were analyzed via HPLC analysis: firstly, it was observed that with the *E. coli* challenge a higher concentration of peptides was obtained, as also observed in our experiments, then it was detected that the most stimulated peptides were a proline-rich peptide, a cecropin-d-like peptide and an anionic peptide-3, this last stimulated also by Gram positive bacterium injection.

Similarly to our work, in Meghashree et al. [83], the infection with *E. coli* and *S. aureus* of *D. melanogaster* and *Drosophila ananassae* larvae showed an increase in protein concentration in hemolymph, and stronger antimicrobial activity, compared to uninfected larvae, in which no inhibition zones in the agar diffusion test were detected. HPLC analysis and the SDS PAGE for high molecular weight proteins showed a differential expression of induced peptides: 3 and 2 peptides were more expressed after *E. coli*/*S. aureus* infection in *D. ananassae* and *D. melanogaster,* respectively, while the SDS PAGE for low molecular weight proteins showed a single protein differentially expressed in both species exclusively after the *E. coli* infection. The LC-MS/MS analysis demonstrated that this protein was a cecropin. As reported by Meghashree et al. [83], the effect of non-induced AMPs is not always easily identifiable: for example, in contrast to our experiments, in experiments on the American Cockroach, *Periplaneta americana*, non-induced hemolymph also did not show any activity against both Gram positive and Gram negative bacteria, whereas induced hemolymph exhibited high activity against *Micrococcus luteus* but less against *E. coli* [84]. However, it is important to notice that, in our experiments, the control sample has a lower antimicrobial effect than the peptide fraction deriving from hemolymph of infected larvae.

The activity of hemolymph both against *E. coli* and *M. flavus* was consistent with what has been reported in the literature, even though not many studies analyze the activity of *H. illucens* hemolymph.

In many cases, an extract from larvae *in toto* is analyzed: for example, in Choi et al. and Auza et al., the antibacterial activity of methanolic extract of *H. illucens* larvae was detected against Gram negative bacteria (*Klebsiella pneumoniae*, *Neisseria gonorrhoeae*, *Shigella sonnei*, *Salmonella typhimurium*, *E. coli* and *P. aeruginosa*) [41,85].

To the best of our knowledge, only a few papers have focused on *H. illucens* hemolymph extract, testing it against few bacterial strains, *E. coli* (strain D31), *M. luteus* and *S. aureus* [55,86]. In Lee *et al*., *H. illucens* larvae were also immunized by *Lactobacillus* species, showing an increase in antimicrobial activity after the infection, as also reported in our paper in which larvae stimulated with Gram positive showed a major reduction in bacteria cell viability against this bacterial group [86]. In Zdybicka-Barabas *et al*., larvae infected with a Gram positive (*M. luteus*) or Gram negative bacterium (*E. coli* D31) and not infected larvae showed good activity exclusively against the Gram positive bacterium strain [55]. Slight activity against *E. coli* was detected exclusively in *E. coli*-challenged larvae, suggesting a higher sensitivity of the *H. illucens* AMPs towards Gram positive bacteria and that also the specific strain that is used for stimulation, and towards which the putative activity is to be detected, is fundamental.

In general, in our experiments the strongest antibacterial activity related to Gram negative or positive bacteria is related to the species used for the infection: indeed, the strongest activity against Gram positive bacteria was recorded in a peptide fraction derived from hemolymph of larvae infected with Gram positive bacteria, with also a lower MIC (1.13 µg) compared to control samples and samples derived from *E. coli* infection, whose MIC is 2.24 µg in both cases. Peptide fraction derived from hemolymph of larvae infected with *E. coli* showed the strongest activity against Gram negative bacteria, with a lower MIC (0.56 µg), compared to control samples and samples derived from larvae infected with *M. flavus*, whose MIC is 4.45 µg and 1.13 µg, respectively. Literature data together with results obtained in our studies encourage testing hemolymph extracts towards different strains of the same bacteria and other bacteria, pathogenic and not.

Further studies are needed, using different bacteria both for the infection that can stimulate the production of different AMPs, and the bacteria against which these peptides could be tested, since the same pool of molecules can have different inhibitory effects. The antimicrobial activity of some peptides (or peptide fractions), indeed, can be displayed in a different way, even on the same bacterial species, but deriving from a different strain. This specific expression could result from the activation of many signaling pathways that control the production of specific defense peptide genes.

The identification of the AMPs of *H. illucens* in the hemolymph and the subsequent production is the first step to find new molecules to use as therapeutic alternatives or in synergy with current antibiotics for applications in the pharmacological and biotechnological fields. Further investigation will include microbiological experiments on the specific peptides differentially expressed and an *in silico* molecular docking against bacterial proteins [44].

## 5. Conclusions

The peptide fraction of hemolymph of *Hermetia illucens* larvae showed antibacterial activity against both Gram negative *Escherichia coli* and Gram positive *Micrococcus flavus* bacteria, depending on used doses and larval infection: although uninfected larvae exhibit antibacterial activity, it can be improved with bacterial infection, inducing a major expression of specific AMPs. After microbiological assays, via mass spectrometry technique we identified 20 AMPs constitutively expressed and 13 inducible by *M. flavus* and *E. coli* infection. The identification of the AMPs of *H. illucens* in the hemolymph could be the starting point to discover alternative molecules to current antibiotics to overcome the problem of antimicrobial resistance.

## Figures and Tables

**Table 1:** Concentrations of the samples obtained via precipitation with organic solvents from plasma extracted from uninfected larvae or larvae infected with *E. coli* or *M. flavus*. Data are expressed as mean ± standard errors of three independent biological replicates.

	Uninfected Larvae	Larvae Infected with *E. coli*	Larvae Infected with *M. flavus*
Precipitation with organic solvents	0.583 ± 0.02 µg/µL	0.739 ± 0.07 µg/µL	0.930 ± 0.03 µg/µL

**Table 2:** Diameters (mm) of inhibition zones formed by peptide fraction, obtained via precipitation with organic solvents, from uninfected larvae, larvae infected with *E. coli* or larvae infected with *M. flavus.* Data are expressed as mean ± standard errors of diameters measured via antibiogram of three independent biological replicates. Different letters indicate significant differences between the same sample against the different strains (capital letters) and among different samples against the same strains (lowercase letters). Data are analyzed with one-way ANOVA and Bonferroni post *hoc test* (*p* value *E. coli* = 0.2690, *M. flavus* = 0.0046) and unpaired *t*-test with Welch’s correction (*p* value uninfected larvae = 0.8113, larvae infected with *E. coli* = 0.3868 and larvae infected with *M. flavus =* 0.0824).

	Uninfected Larvae	Larvae Infected with *E. coli*	Larvae Infected with *M. flavus*
*E. coli*	6.67 ± 1.2 ^aA^	8.67 ± 0.3 ^aA^	8.00 ± 0.5 ^aA^
*M. flavus*	6.33 ± 0.3 ^bA^	8.00 ± 0.6 ^aA^	9.67 ±0.3 ^aA^

## Data Availability

The datasets used and/or analyzed during the current study are available from the corresponding author on reasonable request.

## Supplementary Materials

The following supporting information can be downloaded at: https://www.mdpi.com/article/10.3390/insects14050464/s1, Figure S1. Agar diffusion test of peptide fractions obtained by precipitation with organic solvents, performed against *E. coli* (on the left) or *M. flavus* (on the right). In the figure are reported samples pre- (A) and post- (B) treatment by hexane, as well as the upper fraction possibly containing lipids (C). (a) negative control (H_2_O for A and B, hexane for C); (b) peptide fraction from larvae infected with *E. coli*; (c) peptide fraction from uninfected larvae; (d) peptide fraction from larvae infected with *M. flavus*. The experiments were carried out in triplicate (three independent biological replicates). Table S1a, peptides identified both in infected and uninfected larvae; Table S1b, peptides identified in larvae infected with *E. coli* or *M. flavus*; Table S1c, peptide identified exclusively in larvae infected with *E. coli*; Table S1d, peptides identified exclusively in larvae infected with *M. flavus*. The table reports experimental *m*/*z* value of the peptide, experimental mr value, mascot score, the sequence of identified peptides, the contig code, the amino acid sequence frame (in red the peptides found by LC-MS/MS) and the frame number of the transcriptomic sequence obtain with SEQtools that match with the LC-MS/MS.

## References

[1] Guardabassi L., Kruse H. (2009). Principles of Prudent and Rational Use of Antimicrobials in Animals. Guide to Antimicrobial Use in Animals.

[2] O’Neill J. (2014). Antimicrobial Resistance: Tackling a Crisis for the Health and Wealth of Nations.

[3] Scaglione F. (2012). Motivi di Fallimento di Una Terapia Antimicrobica. Guida Alla Terapia Antimicrobica Nella Pratica Clinica.

[4] Barra A.L.C., Dantas L.d.O.C., Morão L.G., Gutierrez R.F., Polikarpov I., Wrenger C., Nascimento A.S. (2020). Essential Metabolic Routes as a Way to ESKAPE From Antibiotic Resistance. Front. Public Health.

[5] High Levels of Antibiotic Resistance Found Worldwide, New Data Shows. https://www.who.int/news/item/29-01-2018-high-levels-of-antibiotic-resistance-found-worldwide-new-data-shows.

[6] Courvalin P. (2008). Predictable and Unpredictable Evolution of Antibiotic Resistance. J. Intern. Med..

[7] Strelkauskas A., Edwards A., Fahnert B., Pryor G., Strelkauskas J. (2015). Microbiology: A Clinical Approach.

[8] Tonk M., Vilcinskas A., Rahnamaeian M. (2016). Insect Antimicrobial Peptides: Potential Tools for the Prevention of Skin Cancer. Appl. Microbiol. Biotechnol..

[9] Moretta A., Scieuzo C., Petrone A.M., Salvia R., Manniello M.D., Franco A., Lucchetti D., Vassallo A., Vogel H., Sgambato A. (2021). Antimicrobial Peptides: A New Hope in Biomedical and Pharmaceutical Fields. Front. Cell. Infect. Microbiol..

[10] Chernysh S., Gordya N., Suborova T. (2015). Insect Antimicrobial Peptide Complexes Prevent Resistance Development in Bacteria. PLoS ONE.

[11] Neshani A., Zare H., Akbari Eidgahi M.R., Hooshyar Chichaklu A., Movaqar A., Ghazvini K. (2019). Review of Antimicrobial Peptides with Anti-*Helicobacter Pylori* Activity. Helicobacter.

[12] Mangoni M.L., Mcdermott A.M., Zasloff M. (2016). Antimicrobial Peptides and Wound Healing: Biological and Therapeutic Considerations. Exp. Dermatol..

[13] Zharkova M.S., Orlov D.S., Golubeva O.Y., Chakchir O.B., Eliseev I.E., Grinchuk T.M., Shamova O. (2019). Application of Antimicrobial Peptides of the Innate Immune System in Combination with Conventional Antibiotics-a Novel Way to Combat Antibiotic Resistance?. Front. Cell. Infect. Microbiol..

[14] Boman H.G. (1995). Peptide Antibiotics and Their Role in Innate Immunity. Annu. Rev. Immunol..

[15] Teixeira V., Feio M.J., Bastos M. (2012). Role of Lipids in the Interaction of Antimicrobial Peptides with Membranes. Prog. Lipid. Res..

[16] Harris F., Dennison S.R., Singh J., Phoenix D.A. (2013). On the Selectivity and Efficacy of Defense Peptides with Respect to Cancer Cells. Med. Res. Rev..

[17] Gomes B., Augusto M.T., Felício M.R., Hollmann A., Franco O.L., Gonçalves S., Santos N.C. (2018). Designing Improved Active Peptides for Therapeutic Approaches against Infectious Diseases. Biotechnol. Adv..

[18] Chongsiriwatana N.P., Lin J.S., Kapoor R., Wetzler M., Rea J.A.C., Didwania M.K., Contag C.H., Barron A.E. (2017). Intracellular Biomass Flocculation as a Key Mechanism of Rapid Bacterial Killing by Cationic, Amphipathic Antimicrobial Peptides and Peptoids. Sci. Rep..

[19] He J., Starr C.G., Wimley W.C. (2015). A Lack of Synergy between Membrane-Permeabilizing Cationic Antimicrobial Peptides and Conventional Antibiotics. Biochim. Biophys. Acta.

[20] Feng Q., Huang Y., Chen M., Li G., Chen Y. (2015). Functional Synergy of α-Helical Antimicrobial Peptides and Traditional Antibiotics against Gram-Negative and Gram-Positive Bacteria *in Vitro* and *in Vivo*. Eur. J. Clin. Microbiol. Infect. Dis..

[21] Spohn R., Daruka L., Lázár V., Martins A., Vidovics F., Grézal G., Méhi O., Kintses B., Számel M., Jangir P.K. (2019). Integrated Evolutionary Analysis Reveals Antimicrobial Peptides with Limited Resistance. Nat. Commun..

[22] Hollmann A., Martinez M., Maturana P., Semorile L.C., Maffia P.C. (2018). Antimicrobial Peptides: Interaction With Model and Biological Membranes and Synergism With Chemical Antibiotics. Front. Chem..

[23] Borah A., Deb B., Chakraborty S. (2021). A Crosstalk on Antimicrobial Peptides. Int. J. Pept. Res. Ther..

[24] Jenssen H., Hamill P., Hancock R.E.W. (2006). Peptide Antimicrobial Agents. Clin. Microbiol. Rev..

[25] Soltani S., Hammami R., Cotter P.D., Rebuffat S., Said L.B., Gaudreau H., Bédard F., Biron E., Drider D., Fliss I. (2021). Bacteriocins as a New Generation of Antimicrobials: Toxicity Aspects and Regulations. FEMS Microbiol. Rev..

[26] Vallet-Gely I., Lemaitre B., Boccard F. (2008). Bacterial Strategies to Overcome Insect Defences. Nat. Rev. Microbiol..

[27] Hillyer J.F. (2016). Insect Immunology and Hematopoiesis. Dev. Comp. Immunol..

[28] Bulet P., Stocklin R. (2005). Insect Antimicrobial Peptides: Structures, Properties and Gene Regulation. Protein Pept. Lett..

[29] Lavine M.D., Strand M.R. (2003). Haemocytes from *Pseudoplusia Includens* Express Multiple Alpha and Beta Integrin Subunits. Insect. Mol. Biol..

[30] Marmaras V.J., Lampropoulou M. (2009). Regulators and Signalling in Insect Haemocyte Immunity. Cell. Signal.

[31] Fauvarque M.O., Williams M.J. (2011). Drosophila Cellular Immunity: A Story of Migration and Adhesion. J. Cell. Sci..

[32] Scieuzo C., Nardiello M., Farina D., Scala A., Cammack J.A., Tomberlin J.K., Vogel H., Salvia R., Persaud K., Falabella P. (2021). *Hermetia illucens* (L.) (Diptera: Stratiomyidae) Odorant Binding Proteins and Their Interactions with Selected Volatile Organic Compounds: An *In Silico* Approach. Insects.

[33] Scala A., Cammack J.A., Salvia R., Scieuzo C., Franco A., Bufo S.A., Tomberlin J.K., Falabella P. (2020). Rearing Substrate Impacts Growth and Macronutrient Composition of *Hermetia illucens* (L.) (Diptera: Stratiomyidae) Larvae Produced at an Industrial Scale. Sci. Rep..

[34] Franco A., Scieuzo C., Salvia R., Mancini I.M., Caniani D., Masi S., Falabella P. (2022). A Mobile Black Soldier Fly Farm for On-Site Disposal of Animal Dairy Manure. Bull. Insectology.

[35] Scieuzo C., Franco A., Salvia R., Triunfo M., Addeo N.F., Vozzo S., Piccolo G., Bovera F., Ritieni A., di Francia A. (2022). Enhancement of Fruit Byproducts through Bioconversion by *Hermetia illucens* (Diptera: Stratiomyidae). Insect. Sci..

[36] Franco A., Salvia R., Scieuzo C., Schmitt E., Russo A., Falabella P. (2021). Lipids from Insects in Cosmetics and for Personal Care Products. Insects.

[37] Franco A., Scieuzo C., Salvia R., Petrone A.M., Tafi E., Moretta A., Schmitt E., Falabella P. (2021). Lipids from *Hermetia illucens*, an Innovative and Sustainable Source. Sustainability.

[38] Triunfo M., Tafi E., Guarnieri A., Scieuzo C., Hahn T., Zibek S., Salvia R., Falabella P. (2021). Insect Chitin-Based Nanomaterials for Innovative Cosmetics and Cosmeceuticals. Cosmetics.

[39] Triunfo M., Tafi E., Guarnieri A., Salvia R., Scieuzo C., Hahn T., Zibek S., Gagliardini A., Panariello L., Coltelli M.B. (2022). Characterization of Chitin and Chitosan Derived from *Hermetia illucens*, a Further Step in a Circular Economy Process. Sci. Rep..

[40] Guarnieri A., Triunfo M., Scieuzo C., Ianniciello D., Tafi E., Hahn T., Zibek S., Salvia R., de Bonis A., Falabella P. (2022). Antimicrobial Properties of Chitosan from Different Developmental Stages of the Bioconverter Insect *Hermetia illucens*. Sci. Rep..

[41] Choi W.H., Yun J.H., Chu J.P., Chu K.B. (2012). Antibacterial Effect of Extracts of *Hermetia illucens* (Diptera: Stratiomyidae) Larvae against Gram-Negative Bacteria. Entomol. Res..

[42] Park S.I., Kim J.W., Yoe S.M. (2015). Purification and Characterization of a Novel Antibacterial Peptide from Black Soldier Fly (*Hermetia illucens*) Larvae. Dev. Comp. Immunol..

[43] Vogel H., Müller A., Heckel D.G., Gutzeit H., Vilcinskas A. (2018). Nutritional Immunology: Diversification and Diet-Dependent Expression of Antimicrobial Peptides in the Black Soldier Fly *Hermetia illucens*. Dev. Comp. Immunol..

[44] Moretta A., Scieuzo C., Salvia R., Popović Ž.D., Sgambato A., Falabella P. (2022). Tools in the Era of Multidrug Resistance in Bacteria: Applications for New Antimicrobial Peptides Discovery. Curr. Pharm. Des..

[45] Moretta A., Salvia R., Scieuzo C., di Somma A., Vogel H., Pucci P., Sgambato A., Wolff M., Falabella P. (2020). A Bioinformatic Study of Antimicrobial Peptides Identified in the Black Soldier Fly (BSF) *Hermetia illucens* (Diptera: Stratiomyidae). Sci. Rep..

[46] Park S.I., Chang B.S., Yoe S.M. (2014). Detection of Antimicrobial Substances from Larvae of the Black Soldier Fly, *Hermetia illucens* (Diptera: Stratiomyidae). Entomol. Res..

[47] di Somma A., Moretta A., Cané C., Scieuzo C., Salvia R., Falabella P., Duilio A. (2022). Structural and Functional Characterization of a Novel Recombinant Antimicrobial Peptide from *Hermetia illucens*. Curr. Issues Mol. Biol..

[48] Kaczor M., Bulak P., Proc-Pietrycha K., Kirichenko-Babko M., Bieganowski A. (2023). The Variety of Applications of *Hermetia illucens* in Industrial and Agricultural Areas—Review. Biology.

[49] Manniello M.D., Moretta A., Salvia R., Scieuzo C., Lucchetti D., Vogel H., Sgambato A., Falabella P. (2021). Insect Antimicrobial Peptides: Potential Weapons to Counteract the Antibiotic Resistance. Cell. Mol. Life Sci..

[50] Hogsette J.A. (1992). New Diets for Production of House Flies and Stable Flies (Diptera: Muscidae) in the Laboratory. J. Econ. Entomol..

[51] Elhag O., Zhou D., Song Q., Soomro A.A., Cai M., Zheng L., Yu Z., Zhang J. (2017). Screening, Expression, Purification and Functional Characterization of Novel Antimicrobial Peptide Genes from *Hermetia illucens* (L.). PLoS ONE.

[52] Dang X.L., Tian J.H., Yi H.Y., Wang W.X., Zheng M., Li Y.F., Cao Y., Wen S.Y. (2006). Inducing and Isolation of Antibacterial Peptides from Oriental Fruit Fly, *Bactrocera dorsalis* Hendel. Insect. Sci..

[53] Cytryńska M., Mak P., Zdybicka-Barabas A., Suder P., Jakubowicz T. (2007). Purification and characterization of eight peptides from *Galleria mellonella* immune hemolymph. Peptides.

[54] Bradford M.M. (1976). A Rapid and Sensitive Method for the Quantitation of Microgram Quantities of Protein Utilizing the Principle of Protein-Dye Binding. Anal. Biochem..

[55] Zdybicka-Barabas A., Bulak P., Polakowski C., Bieganowski A., Waśko A., Cytryńska M. (2017). Immune Response in the Larvae of the Black Soldier Fly *Hermetia illucens*. Invertebr. Surviv. J..

[56] Butturini E., Gotte G., Dell’Orco D., Chiavegato G., Marino V., Canetti D., Cozzolino F., Monti M., Pucci P., Mariotto S. (2016). Intermolecular Disulfide Bond Influences Unphosphorylated STAT3 Dimerization and Function. Biochem. J..

[57] Fusco S., Aulitto M., Iacobucci I., Crocamo G., Pucci P., Bartolucci S., Monti M., Contursi P. (2020). The Interaction between the F55 Virus-Encoded Transcription Regulator and the RadA Host Recombinase Reveals a Common Strategy in Archaea and Bacteria to Sense the UV-Induced Damage to the Host DNA. Biochim. Biophys. Acta Gene Regul. Mech..

[58] Salvia R., Cozzolino F., Scieuzo C., Grimaldi A., Franco A., Vinson S.B., Monti M., Falabella P. (2022). Identification and Functional Characterization of *Toxoneuron Nigriceps* Ovarian Proteins Involved in the Early Suppression of Host Immune Response. Insects.

[59] Schwarz S., Chaslus-Dancla E. (2001). Use of Antimicrobials in Veterinary Medicine and Mechanisms of Resistance. Vet. Res..

[60] Calza L. (2022). Principi Di Malattie Infettive.

[61] Bassetti M., Righi E. (2013). Multidrug-Resistant Bacteria: What Is the Threat?. Hematol. Am. Soc. Hematol. Educ. Program..

[62] Gordon Y.J., Romanowski E.G., McDermott A.M. (2005). A Review of Antimicrobial Peptides and Their Therapeutic Potential as Anti-Infective Drugs. Curr. Eye Res..

[63] Li Y., Xiang Q., Zhang Q., Huang Y., Su Z. (2012). Overview on the Recent Study of Antimicrobial Peptides: Origins, Functions, Relative Mechanisms and Application. Peptides.

[64] Zhang L., Wu W.K.K., Gallo R.L., Fang E.F., Hu W., Ling T.K.W., Shen J., Chan R.L.Y., Lu L., Luo X.M. (2016). Critical Role of Antimicrobial Peptide Cathelicidin for Controlling *Helicobacter pylori* Survival and Infection. J. Immunol..

[65] Rocha I.F., Maller A., de Cássia Garcia Simão R., Kadowaki M.K., Angeli Alves L.F., Huergo L.F., da Conceição Silva J.L. (2016). Proteomic Profile of Hemolymph and Detection of Induced Antimicrobial Peptides in Response to Microbial Challenge in *Diatraea saccharalis* (Lepidoptera: Crambidae). Biochem. Biophys. Res. Commun..

[66] Sultana A., Luo H., Ramakrishna S. (2021). Harvesting of Antimicrobial Peptides from Insect (*Hermetia illucens*) and Its Applications in the Food Packaging. Appl. Sci..

[67] Van Moll L., De Smet J., Paas A., Tegtmeier D., Vilcinskas A., Cos P., Van Campenhout L. (2022). *In Vitro* Evaluation of Antimicrobial Peptides from the Black Soldier Fly (*Hermetia illucens*) against a Selection of Human Pathogens. Microbiol. Spectr..

[68] Wei L., Mu L., Wang Y., Bian H., Li J., Lu Y., Han Y., Liu T., Lv J., Feng C. (2015). Purification and characterization of a novel defensin from the salivary glands of the black fly, *Simulium bannaense*. Parasit Vectors..

[69] Hwang J.-S., Lee J., Kim Y.-J., Bang H.-S., Yun E.-Y., Kim S.-R., Suh H.-J., Kang B.-R., Nam S.-H., Jeon J.-P. (2009). Isolation and Characterization of a Defensin-Like Peptide (Coprisin) from the Dung Beetle, *Copris tripartitus*. Int. J. Pept..

[70] Dimarcq J.L., Zachary D., Hoffmann J.A., Hoffmann D., Reichhart J.M. (1990). Insect Immunity: Expression of the Two Major Inducible Antibacterial Peptides, Defensin and Diptericin, in *Phormia terranovae*. EMBO J..

[71] Zhou J., Liao M., Ueda M., Gong H., Xuan X., Fujisaki K. (2007). Sequence Characterization and Expression Patterns of Two Defensin-like Antimicrobial Peptides from the Tick *Haemaphysalis longicornis*. Peptides.

[72] Liu Y., Ye N., Chen M., Zhao H., An J. (2020). Structural and Functional Analysis of PGRP-LC Indicates Exclusive Dap-Type PGN Binding in Bumblebees. Int. J. Mol. Sci..

[73] Cobo E.R., Chadee K. (2013). Antimicrobial Human β-Defensins in the Colon and Their Role in Infectious and Non-Infectious Diseases. Pathogens.

[74] Mandrioli M., Bugli S., Saltini S., Genedani S., Ottaviani E. (2003). Molecular Characterization of a Defensin in the IZD-MB-0503 Cell Line Derived from Immunocytes of the Insect *Mamestra brassicae* (Lepidoptera). Biol. Cell..

[75] Yang W., Cheng T., Ye M., Deng X., Yi H., Huang Y., Tan X., Han D., Wang B., Xiang Z. (2011). Functional Divergence among Silkworm Antimicrobial Peptide Paralogs by the Activities of Recombinant Proteins and the Induced Expression Profiles. PLoS ONE.

[76] Jayamani E., Rajamuthiah R., Larkins-Ford J., Fuchs B.B., Conery A.L., Vilcinskas A., Ausubel F.M., Mylonakisa E. (2015). Insect-Derived Cecropins Display Activity against *Acinetobacter baumannii* in a Whole-Animal High-Throughput *Caenorhabditis elegans* Model. Antimicrob. Agents Chemother..

[77] Toro Segovia L.J., Téllez Ramírez G.A., Henao Arias D.C., Rivera Duran J.D., Bedoya J.P., Castaño Osorio J.C. (2017). Identification and Characterization of Novel Cecropins from the *Oxysternon conspicillatum* Neotropic Dung Beetle. PLoS ONE.

[78] Hong S.M., Kusakabe T., Lee J.M., Tatsuke T., Kawaguchi Y., Kang M.W., Kang S.W., Kim K.A., Nho S.K. (2008). Structure and Expression Analysis of the Cecropin-E Gene from the Silkworm, *Bombyx mori*. Biosci. Biotechnol. Biochem..

[79] Silverman N., Paquette N., Aggarwal K. (2009). Specificity and Signaling in the *Drosophila* Immune Response. Invertebr. Surviv. J..

[80] Kleino A., Silverman N. (2014). The Drosophila IMD Pathway in the Activation of the Humoral Immune Response. Dev.Comp. Immunol..

[81] Mak P., Zdybicka-Barabas A., Cytryńska M. (2010). A different repertoire of *Galleria mellonella* antimicrobial peptides in larvae challenged with bacteria and fungi. Develop. Compar. Immunol..

[82] Mastore M., Binda Rossetti S., Giovannardi S., Scarì G., Brivio M.F. (2015). Inducible Factors with Antimicrobial Activity after Immune Challenge in the Haemolymph of Red Palm Weevil (Insecta). Innate Immun..

[83] Meghashree R.N., Nagaraj K. (2021). Characterization of the Immune Induced Antimicrobial Peptide in *Drosophila* melanogaster and *Drosophila ananassae*. EJE.

[84] Basseri H.R., Dadi-Khoen A., Bakhtiari R., Abolhassani M., Hajihosseini-Baghdadabadi R. (2016). Isolation and Purification of an Antibacterial Protein from Immune Induced Hemolymph of American Cockroach, *Periplaneta americana*. J Arthropod-Borne Dis..

[85] Auza F.A., Purwanti S., Syamsu J.A., Natsir A. (2020). Antibacterial Activities of Black Soldier Flies (*Hermetia illucens*. l) Extract towards the Growth of *Salmonella typhimurium*, *E. coli* and *Pseudomonas aeruginosa*. IOP Conf. Ser. Earth Environ. Sci..

[86] Lee K.S., Yun E.Y., Goo T.W. (2020). Antimicrobial Activity of an Extract of *Hermetia illucens* Larvae Immunized with Lactobacillus casei against Salmonella Species. Insects..

